# Comparative Evaluation of Antioxidative and Anti-inflammatory Properties of Acerola-Mediated Silver Nanogel and Copper Oxide Nanogel

**DOI:** 10.7759/cureus.65409

**Published:** 2024-07-26

**Authors:** Burnice Nalina Kumari Chellathurai, Ambalavanan N, Rajeshkumar Shanmugam, Jaideep Mahendra, Uma Sudhakar

**Affiliations:** 1 Department of Periodontology, Meenakshi Ammal Dental College and Hospital, Meenakshi Academy of Higher Education and Research, Chennai, IND; 2 Nanobiomedicine Laboratory, Centre for Global Health Research, Saveetha Medical College and Hospital, Saveetha Institute of Medical and Technical Sciences, Chennai, IND; 3 Department of Periodontology, Thai Moogambigai Dental College and Hospital, Chennai, IND

**Keywords:** green nanosynthesis, anti-oxidative property, vitamin c, malphigian emarginata, antiinflammatory, copper oxide nanoparticles, silver nanoparticles, acerola cherry

## Abstract

Background: The tropical plant acerola of the genus *Malpighia* includes shrubs and trees with fruit that is high in nutrients and bioactive chemicals. Acerola stands out due to its exceptionally high ascorbic acid content, ranging from 1500 to 4500 mg/100 g. Vitamin C intake greatly influences gingival health. The addition of nanoparticles along with vitamin C-rich acerola exhibits high antioxidant and anti-inflammatory properties, thereby positively improving gingival health.

Method: The antioxidant and anti-inflammatory properties of aqueous extracts of the acerola plant (*Malpighia emarginata*) were assessed. Silver nanoparticles (AgNPs) and copper oxide nanoparticles (CuONPs) were synthesized using the aqueous extract of acerola cherry gel by the phytogenic fabrication method. The antioxidant potential of silver and copper nanoparticles was evaluated using 2,2-diphenyl-1-picrylhydrazyl (DPPH), hydrogen peroxide, ferric reducing antioxidant power (FRAP), 2,2'-azino-bis(3-ethylbenzothiazoline-6-sulfonic acid) (ABTS), and nitric oxide scavenging activities.

Results: Increasing concentrations of nanoparticles showed an increase in scavenging activity. Overall, CuONPs and AgNPs exhibited remarkable radical quenching efficacies. The anti-inflammatory effectiveness of CuONPs and AgNPs was monitored, showing suppression of protein denaturation as demonstrated by bovine serum albumin (BSA), egg albumin (EA), and membrane stabilization assays. The results revealed that increasing the doses of CuONPs and AgNPs had a positive impact on the anti-inflammatory activity of the nanoparticles.

Conclusion: The present study revealed that both nanoparticles provided better antioxidant and anti-inflammatory activities. This study also elaborates on the pharmacological potential of both nanoparticles, which could be further explored for application in all healthcare sectors.

## Introduction

The body's intricate biochemical response to pathogenic infection, trauma, toxins, and ultraviolet light is called inflammation. It also serves as a tissue repair mechanism for injured tissues [1]. During the inflammation phase, macrophages release substances like cytokines, process antigens through phagocytosis, and play a crucial role in healing. The synthesis of several inflammatory mediators, such as prostaglandins, cyclooxygenase-2 (COX-2), nitric oxide (NO), tumor necrosis factor-alpha (TNF-α), and interleukin-1 beta (IL-1β) [2], is regulated by macrophages during the inflammation phase. These cytokines are indicators of reactions associated with inflammation.

A wide range of medications, including steroids and nonsteroidal anti-inflammatory drugs (NSAIDs), are used to treat inflammation, which is the root cause of many disorders. Historically, the treatment of inflammation has been associated with phytochemicals such as salicylic acid and aspirin. Plant polyphenols [3] isolated from herbs and their derivatives have also been used to treat inflammatory illnesses. Several medicinal plants, such as *Eucalyptus globulus*, *Thymus vulgaris*, *Mentha longifolia*, *Pycnocycla spinosa*, *Echium amoenum* (borage), and *Mitrephora sirikitiae *[4], are rich in polyphenols and have been used as treatments for inflammatory diseases in recent times.

Reactive oxygen species (ROS) and reactive nitrogen species (RNS) are additional metabolic byproducts that demand oxygen for proper functioning in living cells. These byproducts are highly reactive to cellular and mitochondrial membranes and cause cell damage. ROS, which include singlet oxygen (1O_2_), hydroxyl radicals (OH•), superoxide radicals (O_2_•−), and hydrogen peroxide (H_2_O_2_), are oxygen derivatives. RNS consist of peroxynitrite (ONOO−) and nitric oxide (•NO), which are other hazardous chemicals. The oxidation of biomolecules [5] and the modification of proteins and genes by excess ROS may advance inflammatory disorders. Thus, to stop inflammation, antioxidants must quickly remove RNS and ROS from the body.

Plant extracts are efficient scavengers of free radicals for the quick removal of oxidative stressors. Consequently, a variety of plant-derived extracts can be utilized as medications to modulate chronic inflammation [6] brought on by viral infection and other conditions. *Malpighia emarginata* (acerola), also known as the antilles cherry, is a tropical fruit of the genus *Malpighia* that is native to South and Central America. It is a member of the Malpighiaceae family [7]. Phytochemicals in *Malpighia* include ascorbic acid (AA), carotenoids (CA), and phenolic compounds (PC), which are high in vitamin C. Due to its high vitamin C content, antioxidant potential, and antibacterial properties, the seeds, growl, and biomass produced during the commercial cultivation of acerola provide an abundance of potential for use as natural additives or dietary supplements [8]. Using acerola byproducts, researchers have created several new products, including those with antioxidant and antimicrobial activity, micro- or nanoparticles (NPs) harboring active substances, adsorbents, and precursors of sustainable energy. Nanomaterials' high surface-to-volume ratio gives them unique physicochemical properties, making them ideal for biomedical applications where biological processes occur at nanometer scales. As drug carriers, NPs offer high stability, high biocompatibility, large carrier capacity, the incorporation of both hydrophilic and hydrophobic substances, and multiple administration routes, including oral use. Today, metal NPs can be produced by plant extracts and are applied in many high-tech fields, including medicine for imaging, quicker diagnosis, drug delivery, tissue regeneration, cancer treatments, antioxidants, bactericidal and fungicidal agents, and creation of new therapeutics [9]. A significant area of nanotechnology is the production of NPs employing fungi, bacteria, algae, and plants [10].

Even though the acerola cherry has been the subject of substantial research for diverse applications, an in-depth investigation of its bioactivities is still not entirely uncovered. Therefore, the current study aims to elaborate on the green synthesis of silver nanoparticle (AgNP) and copper oxide nanoparticle (CuONP) gels using an aqueous extract of acerola cherry and to evaluate their antioxidant and anti-inflammatory potential for biomedical research.

## Materials and methods

Preparation of acerola cherry aqueous extract

Acerola powder procured from Brut Appetit (RMCA Ventures, Bangalore, India) is a 100% pure freeze-dried product. Exactly 2 g of the powder was blended in 100 mL of distilled water in a 100 mL beaker and heated on a magnetic plate of a magnetic stirrer operated at 55°C. The mixture was heated for 15 minutes with constant stirring. The content was then filtered using Whatman filter paper to remove the residues. The clear filtrate was allowed to cool to room temperature. Finally, the aqueous extract was maintained in a screw-capped container and used for the phytogenic fabrication of NPs.

Phytogenic designing of CuONP gel

A 30 mM copper sulfate solution was prepared in 50 mL of distilled water. Then, 10 mL of the aqueous extract of acerola cherry was amalgamated with 30 mL of the copper sulfate solution and set for continuous shaking in an orbital shaker for 48 hours at 160-180 rpm. After shaking, the entire solution was centrifuged at 8000 rpm for 15 minutes, and the pellet was collected and washed three times with distilled water to remove impurities. One milliliter of this pellet was then mixed with 5 g of gel, consisting of 2.5 g of carbopol and 2.5 g of carboxymethyl cellulose, to achieve a homogeneous gel form. Finally, the prepared acerola-mediated CuONP gel was stored at 4°C and maintained in a vial for bioactivity screening.

Phytogenic designing of AgNP gel

A 2 mM silver nitrate solution was prepared in 80 mL of distilled water. Twenty milliliters of the aqueous extract of acerola cherry were blended with the silver nitrate solution in an orbital shaker operated at 160-180 rpm for 48 hours. After vigorous shaking, the solution was centrifuged for 15 minutes at 8000 rpm, and the pellet was collected after discarding the supernatant. The adhered impurities in the pellet were removed by repeated cleansing with distilled water. One milliliter of this purified pellet was mixed with 2.5 g of carbopol and 2.5 g of carboxymethyl cellulose to achieve a homogeneous gel form. This prepared acerola-mediated silver nanoparticle (AgNP) gel was carefully stored at 4°C for bioactivity investigations.

Antioxidant assays

Antioxidant effects were assessed using the 2,2-diphenyl-1-picrylhydrazyl (DPPH) assay, hydrogen peroxide (H_2_O_2_) radical assay, ferric reducing antioxidant power (FRAP) assay, nitrogen oxide scavenging activity, and 2,2'-azino-bis(3-ethylbenzothiazoline-6-sulfonic acid) (ABTS) assay for AgNP gel and CuONP gel. Five different concentrations used for all antioxidant assays were 10 μg/mL, 20 μg/mL, 30 μg/mL, 40 μg/mL, and 50 μg/mL.

DPPH Assay

The DPPH experiment was used to assess the scavenging capacity of acerola-mediated AgNP gel and CuONP gel individually in a dose-dependent manner. The respective NPs, along with the DPPH solution, were incubated for approximately 30 minutes in the dark. Then, the absorbance was measured at 517 nm. Vitamin C was used as the standard, and the % inhibition was calculated by the equation:

% inhibition (DPPH) = [100 - (ABS sample - ABS control) × 100)]/ABS control

where ABS sample stands for the absorbance of the sample and ABS control stands for the absorbance of the control.

Hydrogen Peroxide (H_2_O_2_) Radical Assay

The protocol demonstrated by Halliwell and co-workers was applied to assess the antioxidant activity of the acerola-mediated AgNP gel and CuONP gel at five different concentrations (10, 20, 30, 40, and 50 μg/mL). In this assay, the degradation of 2-deoxyribose creates a chromogenic compound (malondialdehyde) by the hydrogen peroxide radical. This color change is directly proportional to the quantity of hydrogen peroxide. Briefly, the reaction mixture (1 mL) consisted of 2-deoxyribose (100 µL, 1 mL) prepared in phosphate buffered saline (PBS) (pH 7.4). To the content was added a diverse range (10-50 μg/mL) of acerola-mediated AgNP gel and CuONP gel. Then, 200 μL ferric chloride (200 mM) and EDTA (1.04 mM) were added along with 100 μL of 1 mM hydrogen peroxide and 100 μL AA. The whole reaction mixture was retained for 1 hour at ambient conditions, and the damage imposed by the free radical (H_2_O_2_) on the substrate (deoxyribose) was checked by measuring the absorbance spectrophotometrically at 532 nm. AA was used for comparison. The inhibitory percentage (%) of degradation of deoxyribose was found by applying the formula [11]:

Inhibition (%) of H_2_O_2_ radical = [Control (A532) - Sample (A660)/Sample (A532)] x 100

where Control (A532) is the absorbance of the control and Sample (A532) is the absorbance of screened NPs. From the formula, the IC50 was determined.

FRAP Assay

As per the standard protocol, the antioxidant impact of acerola-mediated AgNP gel and CuONP gel was performed. For this assay, 3.1 g of 300 mM sodium acetate trihydrate (C_2_H_3_NaO_2_·3H_2_O), acetic acid (16 mL) with pH 3.6, 2,4,6-tripyridyl-s-triazine (TPTZ, 10 mM), ferric chloride hexahydrate (20 mM), and HCl (40 mM) comprised the stock solution. A fresh working solution was made by diluting acetate buffer (25 mL), TPTZ (2.5 mL) solution, and ferric chloride hexahydrate solution (2.5 mL). To this, varying doses (10-50 µg/mL) of acerola-mediated AgNP gel and CuONP gel, along with the FRAP solution (1.5 mL), were added, and the whole content was left to react for five minutes in a dark environment. The intensity of the developed color was measured spectrophotometrically at 593 nm. AA was used as a standard comparative drug. The percentage (%) inhibition was found using the formula [12]:

Inhibition (%) of FRAP radical = [Control (A593) - Sample (A593)/Sample (A593)] x 100

where Control (A593) is the absorbance of the control and Sample (A593) is the absorbance of screened NPs. From the aforementioned formula, the IC50 was determined.

Nitric Oxide Scavenging Activity

The scavenging ability of acerola-mediated AgNP gel and CuONP gel against nitric oxide radicals was ascertained using the Ebrahimzadeh method [13]. Sodium nitroprusside was used to produce nitric oxide, which was then quantified using the Griess reaction. The standard utilized was curcumin, a naturally occurring direct nitric oxide scavenger that prevents the induction of nitric oxide synthase. It reduces the quantity of nitrite produced when oxygen and nitric oxide from sodium nitroprusside combine. The following formula was used to calculate the % antioxidant activity after measuring the absorbance at 596 nm:

% Antioxidant activity = [100 - (ABS sample - ABS control) × 100]/ABS control

where ABS sample is the absorbance of the sample and ABS control is the absorbance of the control.

ABTS Assay

The ABTS quenching assay procedure for AgNPs and CuONP gels was carried out by Janani et al. [14]. A working solution was prepared by blending an equal quantity of 2.4 mM potassium persulfate with a 7 mM ABTS solution. The resultant mixture was subsequently kept at room temperature for 12 hours in a dark environment. Next, 1 mL of the resulting solution was thoroughly mixed with 1 mL of various concentrations (10 μg/mL to 50 μg/mL) of AgNPs and CuONPs. The reacting mixture was determined by spectroscopy at 734 nm six minutes later. The following formula was employed to calculate the percentage (%) of acerola-mediated AgNP gel and CuONP gel capability to scavenge ABTS:

Percentage of inhibition by ABTS = [C(Abs) - S(Abs)]/C(Abs) x 100

where C(Abs) is the control absorbance and S(Abs) is the sample absorbance.

Anti-inflammatory Assessment

To prove the anti-inflammatory potency of acerola-mediated AgNP gel and CuONP gel, an albumin denaturation inhibition assay, bovine serum albumin (BSA) denaturation inhibition assay, and membrane stabilization method were performed as per the documented protocols with minor alterations.

Egg Albumin (EA) Denaturation Inhibition Assay

Briefly, freshly prepared hen egg white albumin (0.2 mL) and PBS (2.8 mL) of pH 6.4 were mixed with different doses (10 μg/mL to 50 μg/mL) of acerola-mediated AgNP gel and CuONP gel, individually. The tube contents were placed in a biological oxygen demand (BOD) incubator at ambient conditions for 15 minutes. Then denaturation was induced by heating the reaction mixture contents at 65°C for five minutes. All the tubes were cooled and the absorbance was measured at 660 nm with the aid of a UV-visible spectrophotometer. Diclofenac sodium, a well-known NSAID, was used as a positive control. Distilled water was used as a blank and dimethylsulfoxide (DMSO) as a control. The protein denaturation inhibition was depicted as percentage (%) inhibition using the formula:

Inhibition (%) of protein denaturation = [Control (A660) - Sample (A660)/Sample (A660)] x 100

where Control (A660) is the absorbance of the control and Sample (A660) is the absorbance of screened NPs. From the aforementioned formula, IC50 was determined [15].

BSA Denaturation Inhibition Assay

The anti-inflammatory potency of the acerola-mediated AgNP gel and CuONP gel was screened using a BSA protein denaturation assay with minor amendments. Precisely, 0.5 mL of various doses (10-50 µg/mL) of CuONP and AgNP gels were separately added into test tubes containing 0.2% of a 0.5% BSA solution (prepared in Tris-buffer, pH 6.8). The mixture was incubated at 37°C for 15 minutes, followed by immersion in a hot water bath set at 72°C for five minutes. Post-incubation, the test tube contents were cooled, and the turbidity acquired due to heat-induced protein precipitate was recorded in a UV-visible spectrophotometer at 660 nm. Diclofenac sodium was used as a positive control. The inhibition percentage (%) was calculated using the following formula:

Inhibition (%) of protein denaturation = [Control (A660) - Sample (A660)/Sample (A660)] x 100

where Control (A660) is the absorbance of the control and Sample (A660) is the absorbance of screened NPs [16].

Membrane Stabilization Method

To execute this anti-inflammatory assay, blood was freshly drawn from healthy individuals and mixed with anticoagulants. Centrifugation was done for 10 minutes at 3000 rpm, and the packed cells were separated and washed using iso-saline. Then, a 10% (v/v) suspension was made using iso-saline. The suspension of HRBC was utilized to assess the anti-inflammatory potential of the NPs. Specifically, a suspension of HRBC (0.5 mL), phosphate buffer (1 mL), and hyposaline (2 mL) were mixed with varying doses (10-50 µg/mL) of acerola-mediated AgNP gel and CuONP gel individually. The entire reaction mixture was left to react for 30 minutes under ambient conditions before centrifugation. The contents were centrifuged at 3000 rpm for two minutes. The supernatant was taken, and the absorbance was checked to identify the hemoglobin (Hb) content using a UV-visible spectrophotometer operating at 560 nm. The inhibitory percentage (%) of hemolysis was calculated using the following formula:

Hemolysis inhibition in % = [Control (A560) - Sample (A560)/Sample (A560)] x 100

where Control (A560) is the absorbance of the control and Sample (A560) is the absorbance of screened NPs [17].

Statistical Execution

All experiments were performed in triplicate, and the values were noted for graph plotting. Data were processed using GraphPad Prism version 8.0.1 (GraphPad Software Inc., La Jolla, California). For the in vitro investigations, including antioxidant and anti-inflammatory activity assessments, significant differences among treatment means were determined using one-way ANOVA with Tukey's post-hoc test (p < 0.05).

## Results

DPPH assay

**Figure 1:**
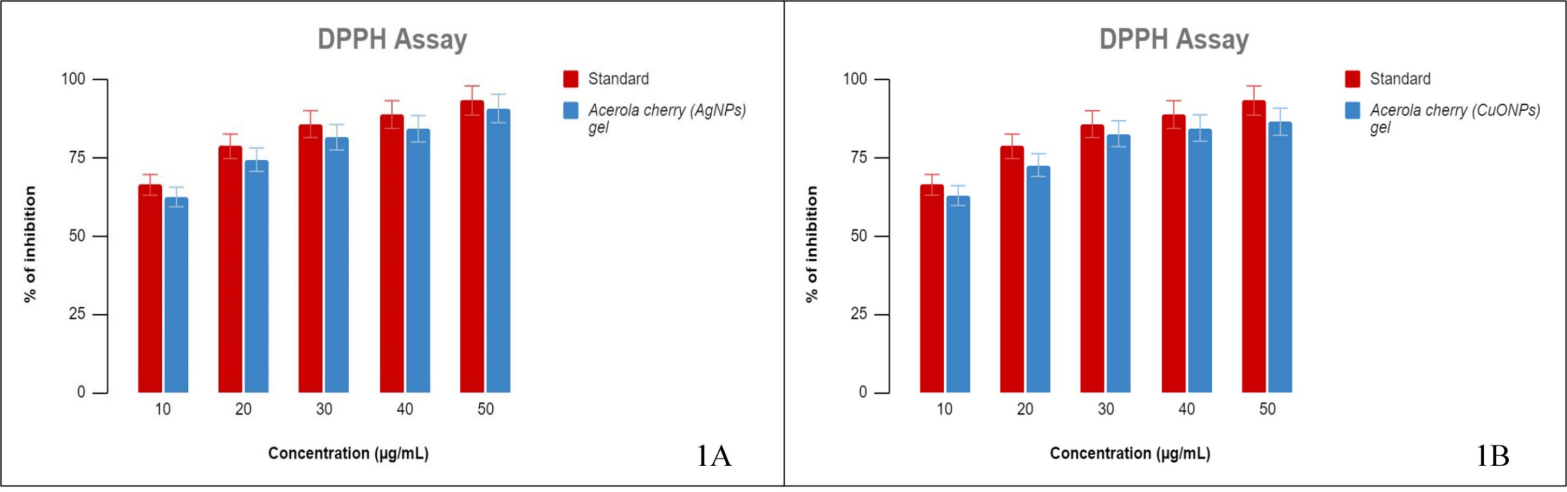
DPPH assay (A) The % inhibition of AgNPs and the standard by DPPH assay; (B) the % inhibition of CuONPs and standard by DPPH assay. DPPH: 2,2-diphenyl-1-picrylhydrazyl, AgNPs: silver nanoparticles, CuONPs: copper oxide nanoparticles.

It was observed that among the five different concentrations of acerola-mediated AgNP gel and CuONP gel tested, the maximum inhibition of 90% and 76%, respectively, was observed at 50 μg/mL. A minimum inhibition of 62% was observed at the lowest concentration of 10 μg/mL, as shown in Figure 1 A and B. The IC50 for the synthesized AgNP gel and CuONP gel was 8.5 μg/mL, indicating their effectiveness in scavenging 50% of the DPPH radicals.

H_2_O_2_ scavenging ability

**Figure 2:**
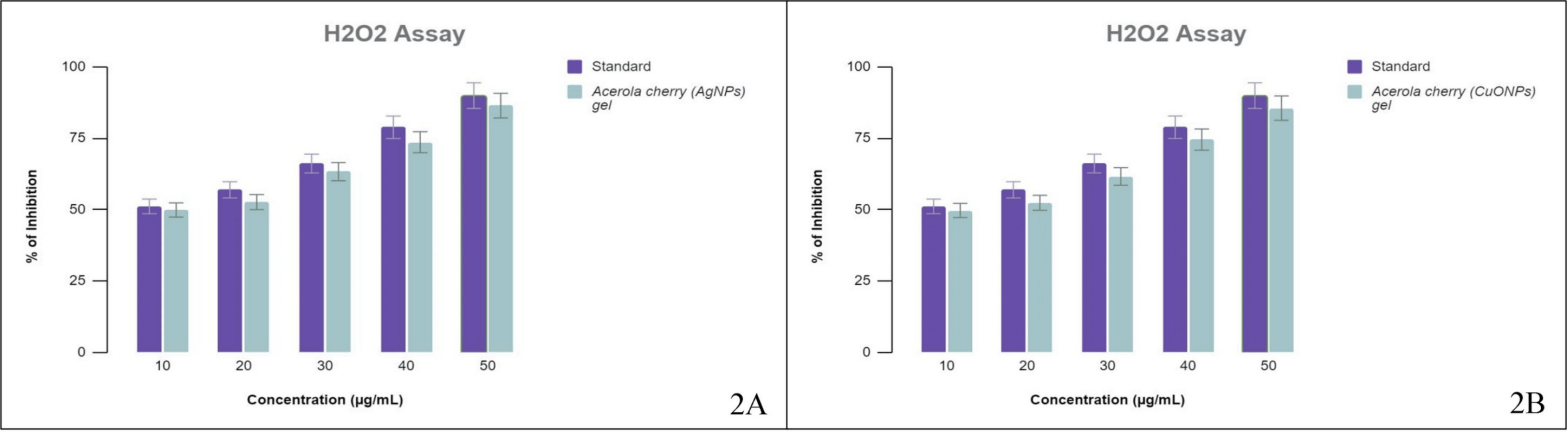
Hydrogen peroxide scavenging ability (A) The % inhibition of AgNPs and the standard (diclofenac sodium); (B) the % inhibition of CuONPs and the standard (diclofenac sodium). AgNPs: silver nanoparticles, CuONPs: copper oxide nanoparticles.

The quenching ability of H_2_O_2_ by acerola-mediated AgNP gel and CuONP gel was concentration-dependent (10-50 μg/mL), as shown in Figure 2 A and B (P < 0.05). The results showed that the % inhibition of acerola-mediated AgNP gel and CuONP gel was 86% and 85%, respectively.

FRAP assay

**Figure 3:**
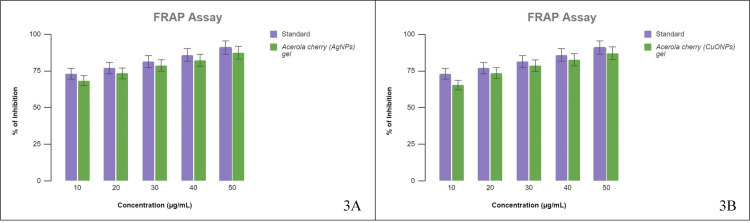
FRAP assay (A) The conversion of Fe_3_^+^ to Fe_2_^+^ by AgNPs and standard; (B) The conversion of Fe_3_^+^ to Fe_2_^+^ by CuONPs and standard. FRAP: ferric reducing antioxidant power, AgNPs: silver nanoparticles, CuONPs: copper oxide nanoparticles.

The capacity of acerola-mediated AgNP gel and CuONP gel to convert Fe_3_^+^ to Fe_2_^+^ in the FRAP experiment varied from 65% to 87% and 68% to 87%, respectively (Figure 3 A and B). The IC50 value was calculated to be 8 μg/mL.

ABTS assay

**Figure 4:**
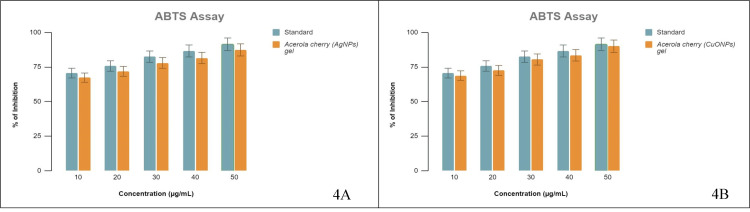
ABTS scavenging activity (A) The dose-dependent % inhibition of AgNPs with the standard BHA; (B) The dose-dependent % inhibition of CuONPs with the standard BHA. ABTS: 2,2'-azino-bis(3-ethylbenzothiazoline-6-sulfonic acid), AgNPs: silver nanoparticles, CuONPs: copper oxide nanoparticles, BHA: butylated hydroxyanisole.

The ABTS•+ radical is a colorful chemical that absorbs at 734 nm. Antioxidant materials react with the ABTS•+ radical, which then transfers electrons to become a non-radical ABTS substance. The study employed spectrophotometric measurement to monitor the decreasing absorbance value at 734 nm and estimated the ABTS•+ radical quenching capacity. The ABTS•+ scavenging activity is commonly used to assess the radical scavenging activities of liquid mixtures, beverages, and pure materials [18]. The ABTS•+ scavenging activity of acerola-mediated AgNP gel and CuONP gel was compared with the standard. The ABTS radical quenching inhibition of acerola-mediated AgNP gel and CuONP gel showed a concentration-dependent manner. At increasing concentrations, there was an evident rise in the reduction of free radicals. According to the findings, at a 50 μg/mL concentration, butylated hydroxyanisole (BHA) achieved 91% inhibition, whereas CuONP gel and AgNP gel achieved 89.9% and 87.33% ABTS•+ radical scavenging activity, respectively (Figure 4 A and B). It was found in this study that CuONPs scavenge ABTS•+ radical better than AgNPs.

NO scavenging ability

**Figure 5:**
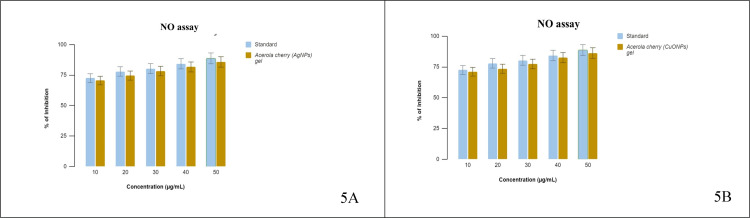
Nitric oxide scavenging activity (A) The % inhibition of AgNPs and standard ascorbic acid; (B) The % inhibition of CuONPs and standard ascorbic acid. NO: nitric oxide, AgNPs: silver nanoparticles, CuONPs: copper oxide nanoparticles.

The potential of the nanoparticles to interact directly with oxygen and nitrogen oxides in the reaction mixture to avert the generation of nitrite allowed scientists to assess their nitric oxide scavenging activity. Excessive production of NO is associated with an array of disorders [19]. The findings of this study illustrate the nitric oxide scavenging capability of the acerola-mediated AgNP gel and CuONP gel (Figure 5 A and B). When compared to conventional AA, the nanoparticles' scavenging activity was reduced.

Anti-inflammatory activity

EA Assay

**Figure 6:**
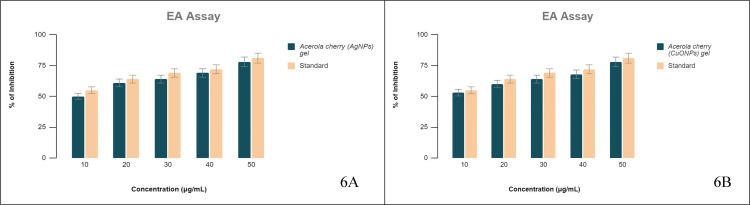
EA assay The % inhibition of (A) AgNPs and (B) CuONPs in a dose-dependent manner in comparison to the standard. EA: egg albumin, AgNPs: silver nanoparticles, CuONPs: copper oxide nanoparticles.

An inexpensive substitute for utilizing the denaturation technique to assess the anti-inflammatory properties of any compound is the EA approach. The notion of the EA denaturation assay includes screening the anti-inflammatory nature of the compounds that would be potent in stabilizing the protein architecture [20] and hindering protein denaturation, which is commonly linked to tissue and cellular inflammation. In the executed assay, acerola-mediated AgNP gel and CuONP gel were screened for anti-inflammatory traits. The results showed that the inhibition of protein denaturation by CuONP gel ranged from 53% to 78% at the screened doses, and for AgNP gel, it ranged from 50% to 78%. The IC50 value of CuONP gel was 9.9 µg/mL, whereas for AgNP gel, it was 10.1 µg/mL. Both the NPs provided dose-dependent protein denaturation inhibition capacity (Figure 6 A and B). The AA standard provided 55% to 81% anti-inflammatory potency with an IC50 of 8.7 µg/mL for CuONP gel, whereas for AgNP gel, it was 56% to 81% with an IC50 dose of 8.1 µg/mL.

BSA Assay

**Figure 7:**
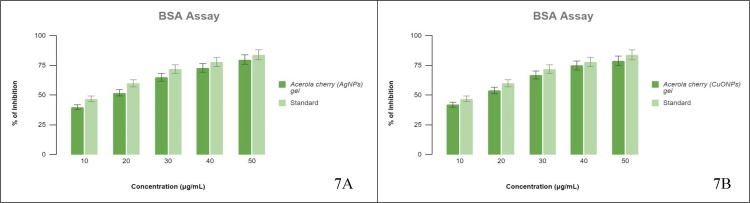
BSA assay The % inhibition by (A) AgNPs and (B) CuONPs in a dose-dependent manner in comparison to the standard. BSA: bovine serum albumin, AgNPs: silver nanoparticles, CuONPs: copper oxide nanoparticles.

Both the NP gels provided dose-mediated inhibitory reactions on the denaturation of BSA. The measured IC50 for CuONP gel was 11.4 µg/mL with 42% to 79% inhibition of protein denaturation, while the commonly prescribed reference drug, AA, showed an IC50 dose of 10 µg/mL with an inhibition percentage ranging from 47% to 84%. The screened AgNP gel provided inhibition of albumin denaturation from 40% to 80% with an IC50 of 7.4 µg/mL (Figure 7 A and B). AA demonstrated an IC50 dose of 6.9 µg/mL with 47% to 84% inhibition. Both the acerola-mediated AgNP gel and CuONP gel provided a dose-dependent protein (albumin) inhibition role along with AA.

Membrane Stabilization Assay

**Figure 8:**
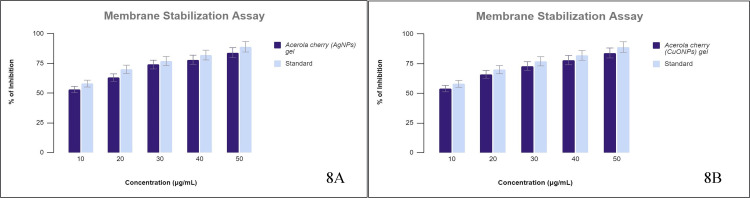
Membrane stabilization assay (A) Membrane stabilization assay of AgNPs. A graph showing the % inhibition by AgNPs in a dose-dependent manner in comparison to the standard. (B) Membrane stabilization assay of CuONPs. A graph showing the % inhibition by CuONPs in a dose-dependent manner in comparison to the standard. AgNPs: silver nanoparticles, CuONPs: copper oxide nanoparticles.

Both the red blood cell membrane of humans and the membrane of lysosomes display similarities in their integrity and structure. This analogy aids in the utilization of HRBC (human red blood cells) to evaluate the membrane-stabilizing potency of the tested compound. The outcome of the assay (Figure 8 A and B) showed that both the acerola-mediated AgNP gel and CuONP gel were active in a dose-dependent manner in inhibiting membrane destabilization. Here, the CuONP gel gave an inhibition range from 54% to 84% with an IC50 dose of 9.1 µg/mL, and the AgNP gel gave an inhibition range from 53% to 84% with an IC50 dose of 8.2 µg/mL. AA provided an IC50 of 8.8 µg/mL for CuONP gel and 7.5 µg/mL for AgNP gel.

## Discussion

The presented data elucidate a significant dose-dependent antioxidant and anti-inflammatory activity of acerola-mediated AgNP gel and CuONP gel, underlining the profound impact of phytochemicals adhered to nanoparticle surfaces. Notably, AgNP gel displayed superior free radical scavenging abilities [21] compared to CuONP gel, which might be attributed to the presence of PC, flavonoids, and alkaloids in the acerola extract forming a protective layer on the nanoparticles, enhancing their electron or hydrogen donation capabilities. The ability to absorb, neutralize, or quench singlet and triplet oxygen are a few of the important factors that contribute to superior antioxidant activity. They are crucial in reducing DPPH and hydrogen peroxide radicals, which is vital in preventing oxidative stress and subsequent cellular damage.

The efficacy of acerola-mediated AgNP gel and CuONP gel in displaying antioxidant and anti-inflammatory activities, as revealed in our study, draws important parallels with previous research, underscoring the potential of nanoparticle-mediated therapies. Notably, our findings resonate with those of Adebayo et al. [22], who reported substantial DPPH radical scavenging activities for Persea americana-mediated AgNPs. This correlation emphasizes the role of biomolecules adhered to nanoparticle surfaces in enhancing antioxidant capabilities, which is crucial for mitigating oxidative stress-induced cellular damage. The AgNP gel fabricated using *Artemisia absinthium*, *Thymus vulgaris*, and *Humulus lupulus *showed significant radical scavenging capacity through the FRAP assay, which supports our data [23].

Moreover, both nanoparticles demonstrated substantial inhibition of protein denaturation, a key marker of anti-inflammatory activity. AgNP gel showed a more pronounced effect in preventing albumin denaturation, with a lower IC50 value compared to CuONP gel. This suggests that AgNPs may offer a stronger therapeutic potential for conditions characterized by inflammation and oxidative stress, such as rheumatoid arthritis. The inhibition of protein denaturation, primarily through the stabilization of albumin structure against external stressors, contributes to their anti-inflammatory efficacy [24,25].

The study also highlighted the role of nanoparticles in membrane stabilization, a critical function in preventing cellular damage during inflammation. AgNP gel was particularly effective in maintaining cell membrane integrity, thereby inhibiting the release of inflammatory mediators. This aligns with previous findings that nanoparticles can interact beneficially with membrane lipids to fortify cell structures against oxidative injury [26]. The results with CuONP gel, though effective, were less pronounced compared to AgNPs, underscoring the potential variability in the therapeutic efficiency of different nanoparticle formulations. In terms of anti-inflammatory properties, both AgNP and CuONP gels demonstrated significant inhibition of protein denaturation, a fundamental marker of inflammation. AgNP gel, in particular, exhibited a potent inhibitory effect on albumin denaturation with a lower IC50 value than CuONP gel, suggesting a stronger potential in therapeutic applications. This observation is supported by previous studies, such as those utilizing green-synthesized nanoparticles from Phoenix dactylifera and Abutilon indicum [22], which similarly highlighted dose-dependent enhancements in antioxidant properties through a redox mechanism.

Moreover, the anti-inflammatory capabilities of the nanoparticle formulations were further evidenced by their ability to stabilize cell membranes, thus preventing the release of inflammatory mediators. This finding aligns with previous research by Naveed et al [27], who observed a comparable inhibition of protein denaturation through green-synthesized AgNPs, thereby substantiating the anti-inflammatory effects of AgNPs. Furthermore, the role of CuONPs in membrane stabilization, as seen in our study, echoes the findings from research using *Mussaenda frondosa* extract [28], where CuONPs demonstrated notable membrane stability. Many experiments and their outcomes on the anti-inflammatory impact of CuO NPs were in congruence with our report [29].

Overall, these findings support the therapeutic potential of nanoparticles in bioactive formulations. The distinct advantages of nanoparticles, including enhanced surface area-volume ratio and the ability to modulate cellular processes at the molecular level, make them promising candidates in the development of advanced therapeutic agents. Limitations of the study included limited scale production and a lack of assessment of the stability of the gels at varying temperatures. Future studies should continue to explore the mechanistic pathways through which these nanoparticles exert their protective effects, with the aim to optimize and tailor nanoparticle-based treatments for specific clinical applications.

## Conclusions

The current study highlights the significant potential of gels containing acerola-mediated AgNPs and CuONPs in reducing and scavenging free radicals. These radicals include DPPH, nitric oxide, and hydrogen peroxide, which are known contributors to oxidative stress and inflammation in the body. By mitigating the presence of these harmful radicals, the gels can effectively prevent inflammation, which is a critical factor in the development of various diseases. The anti-inflammatory properties of these nanoparticles suggest that they can play a crucial role in maintaining overall health, particularly in the oral cavity where oxidative stress is a common issue.

Consequently, the use of silver and copper oxide nanoparticles derived from acerola cherries is being explored for their potential to prevent oral diseases associated with free radicals, with a specific focus on periodontal diseases. The antioxidant and anti-inflammatory properties of AgNPs and CuONPs could provide a novel therapeutic approach to prevent or reduce the severity of these diseases. By incorporating these nanoparticles into oral care products, it may be possible to enhance the maintenance of periodontal tissue health, offering a new avenue for improving oral hygiene and overall well-being.
